# Memory-based variable neighborhood search for green vehicle routing problem with passing-by drivers: a comprehensive perspective

**DOI:** 10.1007/s40747-022-00661-5

**Published:** 2022-02-08

**Authors:** Lei Cao, Chun-ming Ye, Ran Cheng, Zhen-kun Wang

**Affiliations:** 1 School of Business, University of Shanghai for Science and Technology, Shanghai, 200093 China; 2 Guangdong Key Laboratory of Brain-Inspired Intelligent Computation, Department of Computer Science and Engineering, Southern University of Science and Technology, Shenzhen, 518055 China; 3 School of System Design and Intelligent Manufacturing, Department of Computer Science and Engineering, Southern University of Science and Technology, Shenzhen, 518055 China

**Keywords:** Crowdsourcing, Memory programming, City logistics, Variable neighborhood search

## Abstract

A business delivery model with professional vehicles as well as occasional passing-by vehicles is investigated in this paper. The drivers deliver parcels from the distribution center to customers and the passing-by driver can get a certain amount of compensation in return. To give a satisfactory solution from the perspective of platform owner, customers, professional drivers, occasional drivers, and authority, a multi-layer comprehensive model is proposed. To effectively solve the proposed model, we introduce an improved variable neighborhood search (VNS) with a memory-based restart mechanism. The new algorithm is evaluated on instances derived from Solomon’s benchmark and real-life beer delivery instances. Taguchi experiment is used to tune parameters in the proposed VNS, followed by component analysis and real-life experiments. Experimental results indicate that the proposed strategies are effective and the new delivery model in this paper has some advantages over traditional and single-delivery ones from the comprehensive perspectives of stakeholders in the crowdsourcing logistics system.

## Introduction

Urbanization and e-commerce are the keys to drive the strong demand for last-mile delivery and same-day delivery services. The last few decades have seen a great boom in city logistics. More geographic concentration and increasing online orders per person give rise to a great increase in the quantity of the goods to be handled. Especially, e-commerce plays an important role in the current prevention and control of COVID-19 because of its advantage of technology and business model. It is worth mentioning that the COVID-19 may further change the shopping habits of residents. More and more vehicles travelling on urban roads bring lots of negative impacts on health, environment, and safety.

After the Paris Summit in 2015, more and more countries began to pay attention to carbon emissions. To achieve the goal of the Paris Agreement, many countries have taken some effective measures [1], such as legislation, fiscal incentives, carbon tax, and carbon trading, etc. In the field of logistics, environmental protection awareness of residents and new governmental legislation force companies to change their economic-oriented operating strategies.

The rapid development and popularization of mobile and wireless communication technologies may enable achieving some more creative solutions for performing a low-cost and eco-friendly logistics delivery operation. Meanwhile, various new operation modes based on the sharing economy are emerging in many social aspects [2, 3]. Crowdsourcing logistics (CL) mode is produced in the context of the sharing economy, where the crowd can share their excess transport capacity and spare time [4]. Alternative terms crowdshipping, crowdsourcing delivery, crowd logistics, cargo hitching, and collaborative logistics are also found in the realm of the CL. In this paper, we follow the broader definition by Rai et al. [5] who described the CL as “information connectivity enabled marketplace concept that matches supply and demand for logistics services with an undefined and external crowd that has the free capacity with regards to time and/or space, participates voluntarily and is compensated accordingly”.

Alnaggar et al. [6] grouped the crowdsourcing logistics platform (CLP) into two main categories, e-retailers and couriers. As far as we know, for e-retailers, Amazon Flex and Walmart Spark Delivery are currently the only available crowdsourcing delivery platforms in North America. Jingdong Daojia is among the pilot crowdsourcing delivery platforms in China. In contrast, courier crowdsourcing delivery companies are quickly growing in number. Typical companies of this type include Postmates, Deliv, UberEats, Roadie, PiggyBee, DHL MyWays, UberFreight, and Renren Kuaidi [6].

Crowdsourcing logistics promises social, economic, and environmental benefits covering a range of stakeholders. The platforms attempt to achieve a win–win situation by connecting enterprises with part-time individuals who have spare capacity and time. For crowdsourcing logistics enterprises, crowdsourcing delivery can help them reduce logistics costs, especially for last-mile delivery. It also helps satisfy the growing demand for e-commerce logistics, especially during certain e-commerce promotional periods (i.e., Black Friday in North America, Double Eleven in China). In addition to saving of fixed costs, these part-time drivers can work anytime and anywhere, adding flexibility to the delivery process. From an environmental perspective, crowdsourcing delivery may help alleviate the traffic congestion problem and reduce carbon emissions. From a social perspective, crowdsourcing logistics provides employment opportunities for people who have trouble getting a full-time job, or want to earn extra money. Nonetheless, crowdsourcing delivery has some pitfalls and uncertainties. Such service may not be as reliable as those provided by the traditional delivery mode. Crowd drivers are often idle and lack motivation of delivery when the compensation is low, which results in an unguaranteed number of occasional delivery personnels. Moreover, it is a difficult problem to balance compensation and convenience for crowd drivers when CLP owner assigns tasks to them. Thus, companies have to consider the uncertainty as well as the sustainability of the large pool of part-time labor force. Last but not least, some scholars [5, 7, 8] claimed that many factors determine whether crowd logistics has a positive or negative impact on the environment. Thus, the positive environmental impact of crowd logistics is thus up for debate in crowdsourcing delivery.

Generally, the main challenges of crowdsourcing delivery are: (1) The introduction of occasional drivers complicates the neighborhood structures, where moving may occur in professional routes or occasional routes. Furthermore, a new solution may also be obtained by operating customers who belong to different types of routes. (2) The formulated problem is similar to a vehicle routing problem with profit. The difference is that orders should be assigned reasonably to professional drivers or occasional drivers to guarantee service level instead of cost or benefit firstly. An efficient encoding method is needed to represent assignment and routing both in the professional routes and occasional routes. (3) There are several stakeholders in the crowdsourcing logistics system, the interests of which are conflicting. It is a tricky problem to balance these interests. (4) In terms of the tradeoff between exploration and exploitation for neighborhood-based search algorithms, the memory representation mechanism requires elaborate modifications to work efficiently.

To meet the above challenges, a comprehensive model is proposed to consider the satisfaction of stakeholders in the crowdsourcing logistics system, and an enhanced variable neighborhood search algorithm is applied to solve that model.

The remainder of this paper is organized as follows. Related works and contributions gives the literature review of vehicle routing problem with occasional (crowd) drivers and contributions of this paper. In Problem description and formulation, green vehicle routing problem with passing-by drivers and time windows is described in detail. Solution approach gives a specific designed variable neighborhood search algorithm for the model in this paper. Instance generation and Computational experiments are about instance generation, parameter tuning, and experiments. Conclusion ends this paper with conclusions and future work.

## Related works and contributions

As an emerging logistics operation mode, the crowdsourcing logistics is not only applied in the business community, but also increasingly favored by the academic community. Related academic research mainly focuses on matching, routing, driver scheduling, and compensation [6]. In this section, we only review literature related to operations research (OR), especially the routing community, that explicitly addresses crowdsourcing delivery.

Both Amazon and Walmart consider using crowdshipping, who employ crow already on the road to pick up goods from their stores and transport goods to their customers. The concept of crowdshipping proposed by Walmart [9] has spawned a series of quantitative studies and mathematical models. In a study done by Archetti et al. [10], a static deterministic model was introduced called the vehicle routing problem with occasional drivers (VRPOD). In this variant of the vehicle routing problem (VRP), the company owns a fleet of professional vehicles that can only perform closed routes, and a set of static and deterministic occasional drivers (ODs) that can only visit one customer before they head to their intended destination without returning to the depot. Since then, other variants of the VRPOD have been explored.

Based on the research of Archetti et al. [10], Macrina et al. [11] introduced time windows for both customers and ODs and allowed multiple deliveries in their mathematical model. The work of Macrina et al. [11] was recently extended by Macrina et al. [12], who introduced the transshipment nodes in the service network. Dahle et al. [13] extended the model proposed in [10] to a version of pickup and delivery routing problem with time windows and ODs. [14] is among few studies about green vehicle routing problems with ODs, focusing on the environmental impacts of the three types of vehicles.

All the studies above model crowdsourcing logistics as a static system, neglecting the evolution and uncertainty of information. Recently, several researchers have started to study crowdsourcing logistics in the context of stochastic or uncertain parameters. Dahle et al. [15] considered the availability of ODs as an uncertain parameter and assumed stochastic information of ODs’ appearance was known in advance. By comparing with deterministic model, they highlighted that the use of a stochastic formulation might lead to more profitable solutions in terms of saving costs. Dayarian and Savelsbergh [16] considered a VRPOD variant with dynamic customers requests. They developed and compared two rolling horizon dispatching methods. Considering ODs may reject assignments, Gdowska et al. [17] gave each pair (OD-request) a random probability to study dynamic VRPOD. Their results pointed out the need of defining a dynamic compensation scheme for ODs. More recently, Archetti et al. [18] investigated a vehicle routing problem in which customer requests were either known in advance, or arrive online during the distribution process.

Most studies about VRPOD highlight the advantage of using crow in terms of reduction of operational costs. A few aforementioned studies have been devoted to a comprehensive consideration of economic cost, stakeholders’ satisfaction, and external cost (e.g., environmental cost) in crowdsourcing model. Actually, high satisfaction of stakeholders is the guarantee for the reliable and orderly operation of the novel logistics system. At least six stakeholders are directly related to crowdsourcing logistics: senders who need the service of shipments, customers who receive the goods, platform providers who coordinate supply and demand, logistics service providers (LSPs) who provide traditional transport services, the crowd, and authority (or society). Rai et al. [19] studied stakeholders in crowdsourcing logistics system quantificationally, indicating that usage of current platform results in higher external transport costs and thus a higher environmental impact, when compared to traditional goods delivery mode.

In addition to OR-based combinatorial optimization theories, there are many other theories that can support a more eco-friendly and satisfactory crowd logistics system design, such as green economy, consumer utility theory, and equity theory. For the sake of concise and clear literature review structure, the related theory is applied in Problem description and formulation.

The variable neighborhood search algorithm (VNS) is an effective algorithm for solving combinatorial optimization problems. Various strategies were proposed to improve performance of classic VNS. Stenger et al. [20] proposed an adaptive variable neighborhood search algorithm. The algorithm selected a shaking solution from a solution pool, and applied the selection method of removal and insertion operators in the adaptive large neighborhood search algorithm. Kalayci [21] used the ant colony algorithm to restart VNS for the sake of obtaining a high-quality initial solution. Based on historical search success rate, Li et al. [22] proposed a novel method to select neighborhood structure. It is worth mentioning that reinforcement learning is another research line of neighborhood structure selection, and Silva et al. [23] are among the rare studies related to this methodology. To accelerate the speed of local search phase, Thevenin [24] applied a quick evaluation method of solution. Besides, memory mechanism is usually used to enhance VNS (e.g., Pacheco et al. [25] and Li et al. [22]).

To sum up, the main scientific contributions of this work are: (1) To improve the quality and reliability of service of the whole crowd logistics system from a comprehensive perspective of stakeholders. Concerns of different stakeholders in this paper are summarized in Table 1. (2) To propose a variant of VRPOD which allows the passing-by drivers to deliver parcels and multi-delivery. Most literature assumes that crowdsourcing drivers are in-store customers, but this idea was hard to implement in practice. Passing-by drivers are seen as crowd delivery drivers in this work. Actually, in-store customers are one kind of passing-by drivers. To increase willingness to act of ODs, we allow them to deliver more than one parcel, which is different from [10]. (3) To propose a memory-based variable neighborhood search algorithm. Currently, there are a few meta-heuristic algorithms proposed for solving VRPOD, such as [10, 12, 26]. This work enhances the basic variable neighborhood search by introducing memory into restart phase, which gives one certain direction for developing neighborhood-based algorithms.

**Table 1:** Concerns of Stakeholders

Stakeholder	Concerns(s)
CLP owner	Operation cost
	Service level
	Sufficient supply of ODs
	Rate of customer covered
Occasional driver	Compensation
Professional driver	Equality
Customer	Delivery in time window
Authority	Carbon emission

## Problem description and formulation

It is worth mentioning that the research background of this paper is urban commodity transportation, and the only sender has the ownership of CLP. To reduce operating costs and improve delivery efficiency, a CLP owner uses its own professional drivers and occasional drivers to complete the delivery of orders. An occasional driver can be seen as a taxi or truck driver passing by the distribution center. Both the professional drivers and the occasional drivers pick up the goods from the distribution center and deliver them to the customers. The professional driver needs to return to the distribution center, while the occasional driver goes directly to his or her destination after completing the delivery task. At the same time, occasional drivers can get corresponding compensation according to the task attributes. The scheduling plan needs to meet the constraints of service level, occasional driver action, vehicle capacity, while considering customer covered rate, usage rate and equity of professional routes, and operating cost which includes fuel cost, overtime wage, and carbon emission cost. The problem in this paper is named as the green vehicle routing problem with partial crowdsourcing and participants’ satisfaction (GVRP-PCPS). To clarify the scope of application of this study, the following specific assumptions are made:

### Assumption 1

: occasional drivers can participate in one or more deliveries if the compensation meets their expectations;

### Assumption 2

: where the en-route occasional driver receives pushed notifications and his or her destination is known by the CLP owner;

### Assumption 3

: compensation of crowd delivery task is proportional to the sum of distance between distribution center and customer location in the occasional driver route;

### Assumption 4

: all vehicles in the crowdsourcing logistics system are homogeneous, which means that they have the same capacity and velocity;

### Assumption 5

: the loaded quantity of goods at the distribution center is equal to the total of customer demands in each route.

In Section “Notation and Symbol”, notations for the mathematical model are given. The interests of stakeholders are analyzed in Subsection “Stakeholders’ satisfaction and optimized objectives”, which is applied to formalize the objectives in Subsection “Mathematical model for GVRP-PCPS”.

**Table 2:** Set and its description

Symbol	Description
*C*	Set of customers
*A*	Set of all arcs
*N*	Set of all nodes
*S*	Set of occasional driver’ origins
*V*	Set of occasional drivers’ destinations
*K*	Set of occasional vehicles
$P_u$	Set of used professional vehicles
*P*	Set of professional vehicles

### Notation and symbol

**Table 3:** Constant and its description

Symbol	Description
*Q*	Capacity of vehicle
$\alpha$	A large number in mathematic model
$R^S$	Minimal action cost
$R^V$	Variable cost coefficient
$\lambda$	Compensation coefficient
$w_1$	Fuel price
$w_2$	Carbon tax price
$w_3$	Overtime wage per unit time
$E_f$	Carbon emission conversion factor
$\mu _0$	Fuel consumption rate of empty vehicle
$\mu ^{\text {*}}$	Maximum fuel consumption rate
*Sal*	Basic salary per day of professional drivers
BT	Basic work time per day
DT	Maximum work time per day
SL	Designed service level of CLP

GVRP-PCPS is modeled on a complete graph $G=(N,A)$, where *N* is the set of nodes and *A* is the set of arcs. Let *C* be the set of customers. Let *P* be the set of available professional vehicles. Let *K* be the set of occasional vehicles in the crowdsourcing logistics system. Let $S=\{{{s}_{k}}\}$ the set of locations where ODs receive crowd orders, and $V=\{{{v}_{k}}\}$ the set of ODs’ destinations. We define the node set as $N=C\bigcup \{0\}\bigcup S\bigcup V$. 0 represents the depot (distribution center) where the professional drivers leave and return. Each arc $(i,j)\in A$ has a cost ${{c}_{ij}}$, a travel time ${{t}_{ij}}$, and a distance ${{d}_{ij}}$ between node *i* and node *j*. Note that ${{c}_{ij}}$, ${{t}_{ij}}$, and ${{d}_{ij}}$ satisfy the triangle inequality. Each node $i\in C\bigcup \{0\}$ has a time window $[{{e}_{i}},{{l}_{i}}]$, and each customer $i\in C$ has a demand ${{q}_{i}}$ and service time $se{{r}_{i}}$. *Q* is the capacity of the vehicles. Let ${{x}_{ij}}$ be a binary variable equal to 1 if and only if a professional vehicle traverses arc (*i*, *j*), and $r_{ij}^{k}$ a binary variable equal to 1 if and only if $k\in K$ traverses arc (*i*, *j*). For each node $i\in C\bigcup \{0\}$, let ${{y}_{i}}$ be the available capacity of a professional vehicle when it leaves from the node, and ${{y}_{ij}}$ the mass of parcels transported by professional vehicle in arc (*i*, *j*). Let $a_{i}^{k}$ be the available capacity of the vehicle associated with $k\in K$ after visiting customer $i\in C$, and $a_{ij}^{k}$ the mass of parcels transported by occasional vehicle $k\in K$ in arc (*i*, *j*). For each node $i\in C\bigcup \{0\}$, let ${{arr}_{i}}$ be the arrival time of a professional vehicle at customer $i\in C$. Let $f_{i}^{k}$ indicate the arrival time of vehicle $k\in K$ at customer $i\in C$. All parameters and variables used in Section 3 are shown in Tables 2, 3, 4.

**Table 4:** Variable and its description

Symbol	Description
$c_{ij}$	Travel cost of arc $\left( i,j \right)$
$t_{ij}$	Travel time of arc $\left( i,j \right)$
$d_{ij}$	Distance of arc $\left( i,j \right)$
$[e_i,l_i]$	Time window of customer *i*
$[EST_i,LST_i]$	Acceptable time window of customer *i*
$q_i$	Demand of customer *i*
$ser_i$	Service time of customer *i*
$\gamma _i$	Sensitivity coefficients of customer *i*
$x_{ij}$	A binary variable equal to 1 if and only if arc $(i,j) \in A$ is traversed by a professional vehicle
$r_{ij}^{k}$	A binary variable indicating if arc (*i*, *j*) is traversed by a occasional vehicle $k\in K$
$y_i$	Decision variable specify the available capacity of he professional vehicle after visiting customer *i*
$y_{ij}$	Mass of goods transported by professional vehicle in arc (*i*, *j*)
$a_{i}^{k}$	Available capacity of the vehicle associated with $k\in K$ after visiting customer $i \in C$
$a_{ij}^{k}$	Mass of parcels transported by occasional vehicle $k \in K$ in arc (*i*, *j*)
$arr_{i}$	Arrival time of a professional vehicle at customer $i \in C$
$f_{i}^{k}$	Arrival time of $k\in K$ at customer $i \in C$
$\rho _{ij}$	Fuel consumption rate of arc (*i*, *j*)
$\delta _k$	Binary decision variable indicating if occasional $k\in K$ is employed
$p_{ik}$	Compensation of $k \in K$ if customer is served by *k*
$\beta _{ik}$	Binary decision variable indicating if customer *i* is served by $k\in K$
$C_S$	Set of the served customers
$FAI_p$	Fairness coefficient of professional driver $p\in P$
$overload_{k}$	$overload_{k}$ means the overload of $k\in P\cup K$
$W{{T}_{k}}$	Duration of $k\in P\cup K$

### Stakeholders’ satisfaction and optimized objectives

The satisfaction of CLP owner, customer, occasional driver (OD), and professional driver (PD) and authority is included in our model. The CLP owner who requires goods to be transported wants to reduce operation cost and deliver goods on time through partial crowd delivery mode, while authorities are likely to support solutions enhancing the economic vitality of the city, or solutions with environmental benefits such as a decrease in congestion. Stakeholders’ satisfaction in CLP can be divided into service type, cost type, and balance type. Covered customer rate and service level are significant factors for sustainability of CLP. To describe optimized objectives easily in the following section, we define *uncovered customer rate* (*UCR*):

1 $$\begin{aligned} UCR=1-\frac{|C_{S} |}{|C |}, \end{aligned}$$

where $C_{S}$ means the set of served customers.

*Service level*. In the general vehicle routing problem with time windows, the time of obtaining service for a certain customer is in the determined range $[{{e}_{i}},{{l}_{i}}]$, which means that the service level is good(1), otherwise it is bad (0). In real-life transportation problems, however, the time windows may be violated out of several practical considerations. A little bit deviation from the specified time window is acceptable to customers. We name the wider time window $[ES{{T}_{i}},LS{{T}_{i}}]$ as an acceptable time window of customer *i*. Thus, the service level cannot be described by two states. Fuzzy set theory is a strong tool to describe personal subjective feelings [27], which was applied in some routing problems with time window constraint [28, 29].

If the time when the professional or occasional driver arrives in the location of customer *i* is earlier than the claimed earliest service time ${{e}_{i}}$, he or she should wait at customer *i* until the time window is open. Therefore, arriving early has almost no effect on customer satisfaction. Considering the difference in sensitivity of customers to tardiness, we distinguish customers by sensitivity coefficients ${{\gamma }_{i}}$. The customer service level associated with time window is characterized by fuzzy membership functions based on fuzzy set theory. ${{arr}_{i}}$ can be replaced by $f_{i}^{k}$ in Formula 2 to describe the service level of the occasional driver

2 $$\begin{aligned} U\left( arr_{i}\right) = {\left\{ \begin{array}{ll}1 &{} {\text {arr}}_{i} \in \left[ 0, l_{i}\right] \\ \left( \frac{L S T_{i}-arr_{i}}{L S T_{i}-l_{i}}\right) ^{\gamma _{i}} &{} {\text {arr}}_{i} \in \left( l_{i}, L S T_{i}\right] \\ 0 &{} {\text {arr}}_{i} \notin \left[ 0, L S T_{i}\right] .\end{array}\right. } \end{aligned}$$

Operating cost includes fuel cost of PD routes *TFC*, carbon emission cost *TFEC*, compensation of ODs *ODS*, and overtime wage of PD routes $O{{W}_{k}}$. According to [30], the fuel consumption per unit distance has a linear relationship with the load. We use this method to calculate fuel consumption rate in *TFC* and *TCEC*, as is shown in Formula 3

3 $$\begin{aligned} {{\rho }_{ij}}={{\mu }_{0}}+\frac{{{\mu }^{*}}-{{\mu }_{0}}}{Q}{{y}_{ij}}, \end{aligned}$$

where ${{\rho }_{ij}}$ means fuel consumption rate of arc (*i*, *j*); ${{\mu }_{0}}$ means fuel consumption rate of empty vehicle; $\mu ^*$ means fuel consumption rate of vehicle with full capacity.

*Fuel cost of PDs* routes which is related to arcs traversed, load in each traversed arc, and fuel price

4 $$\begin{aligned} TFC={{w}_{1}}\sum \limits _{(i,j)\in A}{{{d}_{ij}}{{\rho }_{ij}}{{x}_{ij}}}, \end{aligned}$$

where ${{w}_{1}}$ means the fuel price.

*Carbon emission cost* which includes direct carbon emission cost incurred by PD vehicle(s) and incremental carbon emission cost incurred by OD vehicle(s) because of detour(s)

5 $$\begin{aligned} \begin{aligned} TCEC=&{{w}_{2}}{{E}_{f}}(\sum \limits _{(i,j)\in A}{{{d}_{ij}}{{\rho }_{ij}}{{x}_{ij}}}+ \sum \limits _{k\in K}{\sum \limits _{(i,j)\in A}{{{\delta }_{k}}\rho _{ij}^{k}r_{ij}^{k}{{d}_{ij}}}}\\&-\sum \limits _{k\in K}{{{\delta }_{k}}\rho _{{{s}_{k}}{{v}_{k}}}^{k}{{d}_{{{s}_{k}}{{v}_{k}}}}}), \end{aligned} \end{aligned}$$

where ${{w}_{2}}$ means carbon emission tax price; ${{E}_{f}}$ means carbon emission conversion factor; $\delta _k$ means binary decision variable indicating if occasional $k\in K$ is chosen; $\rho _{ij}^{k}$ means fuel consumption rate of vehicle $k\in K$ in arc (*i*, *j*).

*Compensation of ODs* is

6 $$\begin{aligned} ODS=\sum \limits _{i\in C}{\sum \limits _{k\in K}{{{p}_{ik}}{{\beta }_{ik}}}}=\sum \limits _{i\in C}{\sum \limits _{k\in K}{{\lambda {d}_{0i}}{{\beta }_{ik}}}}, \end{aligned}$$

where ${p}_{ik}$ means the compensation when vehicle $k\in K$ is assigned to customer $i\in C$; ${\beta }_{ik}$ means binary decision variable indicating if customer *i* is served by $k\in K$. $\lambda$ means compensation coefficient.

*Overtime wage of PDs* is

7 $$\begin{aligned} OW=w_{3}\underset{p}{\sum }(WT_{p}-BT). \end{aligned}$$

We use the inequality 8 to describe the phenomenon when occasional drivers make decision

8 $$\begin{aligned} \sum \limits _{i}{{{p}_{ik}}}\ge R_{k}^{S}+R_{k}^{V}({{d}_{{{s}_{k}}0}}+{{d}_{0{{i}_{1}}}}+\cdots +{{d}_{{{i}_{end}}v_k}}-{{d}_{0{{v}_{k}}}}),\nonumber \\ \end{aligned}$$

where $R_{k}^{S}$ means to minimal action cost; $R_{k}^{V}$ means to variable cost coefficient; $p_{ik}$ means the compensation after occasional vehicle $k \in K$ deliver the goods of customer $i \in C$. In this paper, we assume that occasional drivers are homogeneous.

The left side of the inequality 8 is the CLP’s compensation function. The right side of the inequality is participation threshold function of occasional driver. The first-to-second items of participation threshold function are minimal action cost and variable action cost.

For the sake of reducing turnover rate, it is necessary to ensure that the scheduling plan is attractive to professional drivers. Equity is measured by comparing the ratio of contributions (or costs) and benefits (or rewards) for each person. We define $FA{{I}_{p}}$ as the fairness coefficient of professional driver *p*. By comparing $FA{{I}_{p}}$ with other drivers, each professional driver ${{p}_{0}}\in P$ has a feeling of unfairness $\underset{p}{\mathop {\max }}\,(FA{{I}_{p}}-FA{{I}_{{{p}_{0}}}},0)$.

9 $$\begin{aligned} FA{{I}_{p}}=\frac{Sal+{{w}_{3}}(W{{T}_{p}}-\mathrm {BT})}{W{{T}_{p}}}, \end{aligned}$$

where *Sal* means the basic salary per day; $w_3$ means to overtime wage per unit time; $WT_{p}$ is the duration of $p\in P$; BT is the basic worktime per day.

Thus, *unfairness* for PDs’ workload is

10 $$\begin{aligned} UF=\frac{\text {1}}{|P_u |}\sum \limits _{{{p}_{1}}}{\sum \limits _{{{p}_{2}}}{\max (FA{{I}_{{{p}_{\text {2}}}}}-FA{{I}_{{{p}_{\text {1}}}}},0)}}, \end{aligned}$$

where $P_{u}$ means the set of used professional vehicles.

Self-own vehicles are asset-heavy, and it is necessary to improve the usage rate of professional vehicles. Usage rate *UR* is used to describe the status of PDs in the schedule plan

11 $$\begin{aligned} UR=\frac{|P_{u} |}{P}+\underset{p\in P_{u}}{\sum }WT_{p}/BT. \end{aligned}$$

Occasional drivers’ participation behavior is mainly stimulated by external factors such as money and material. If the compensation offered by company is higher than stimulation threshold, occasional driver is willing to participate in the delivery task. However, higher compensation means more cost for company, which is against the original intention of using crowd. Naturally, when the compensation is slightly larger than the stimulation threshold of occasional driver, it is more economical for the enterprise.

### Mathematical model for GVRP-PCPS

Multi-objective optimization techniques such as lexicographic goal programming (LGP) are more appropriate for GVRP-PCPS solving, because CLP owner has obvious preference when he or she makes decision. The goals set in order of importance can be defined as follows:

1. maximize the number of customers served by PDs and ODs.
2. maximize the usage rate of professional vehicles.
3. minimize the unfairness of PD routes.
4. minimize the cost of operating cost.

Satisfaction of customers served must be more than the service level designed by CLP owner in advance, which is added to constraints. In addition, OD action constraint must be met in the whole optimization process. According to descriptions above, the objective of GVRP-PCPS can be formulated as follows:

12 $$\begin{aligned}&Minimize:UCR \end{aligned}$$

13 $$\begin{aligned}&Minimize:-UR \end{aligned}$$

14 $$\begin{aligned}&Minimize:UF \end{aligned}$$

15 $$\begin{aligned}&Minimize:TFC+TCEC+ODS+OW. \end{aligned}$$

Besides Formula 8, other constraints of GVRP-PCPS are formulated as follows. Formula 16–Formula 24 are linked to the professional vehicles. Formula 25–Formula 35 are linked to the occasional vehicles. Formula 37–Formula 40 are related to decision variables

16 $$\begin{aligned}&\sum \limits _{j\in C}{{{x}_{ij}}}-\sum \limits _{j\in C}{{{x}_{ji}}}=0,\ \ \ \ \forall i\in C \end{aligned}$$

17 $$\begin{aligned}&\sum \limits _{j\in C}{{{x}_{\text {0}j}}}-\sum \limits _{j\in C}{{{x}_{j\text {0}}}}=0 \end{aligned}$$

18 $$\begin{aligned}&{{y}_{j}}\ge {{y}_{i}}+{{q}_{j}}{{x}_{ij}}-Q(1-{{x}_{ij}}),\ \ \ \ \forall i\in C\bigcup \{0\}, \nonumber \\&\forall j\in C \end{aligned}$$

19 $$\begin{aligned}&{{y}_{0}}\le Q \end{aligned}$$

20 $$\begin{aligned}&\begin{array}{l} {{arr}_{j}}\ge {{arr}_{i}}+se{{r}_{i}}+{{t}_{ij}}{{x}_{ij}}-\alpha (1-{{x}_{ij}}),\\ \forall i\in C\bigcup \{0\}, \forall j\in C \end{array} \end{aligned}$$

21 $$\begin{aligned}&U(arr_{i})\ge SL,\quad i\in C_{S} \end{aligned}$$

22 $$\begin{aligned}&WT_{p}\le DT,\quad p\in P \end{aligned}$$

23 $$\begin{aligned}&{{arr}_{0}}=0 \end{aligned}$$

24 $$\begin{aligned}&\sum \limits _{j\in C}{{{x}_{0j}}}\le |P| \end{aligned}$$

25 $$\begin{aligned}&\begin{array}{l} \sum _{j \in C \cup \{0\} \cup \left\{ v_{k}\right\} } r_{i j}^{k}-\sum _{h \in C \cup \{0\} \cup \left\{ s_{k}\right\} } r_{h i}^{k}=0, \\ \forall i \in C \bigcup \{0\}, k \in K \end{array} \end{aligned}$$

26 $$\begin{aligned}&\sum \limits _{j\in C\bigcup \text { }\!\!\{\!\!\text { }{{\text {v}}_{k}}\text { }\!\!\}\!\!\text { }}{r_{{{s}_{k}}j}^{k}}-\sum \limits _{j\in C\bigcup \text { }\!\!\{\!\!\text { }{{\text {s}}_{k}}\text { }\!\!\}\!\!\text { }}{r_{j{{v}_{k}}}^{k}}=0,\ \ \ \ \forall k\in K \end{aligned}$$

27 $$\begin{aligned}&\sum \limits _{k\in K}{{{r}_{{{s}_{k}}0}}}\le |K| \end{aligned}$$

28 $$\begin{aligned}&\sum \limits _{j\in N}{r_{{{s}_{k}}j}^{k}}\le 1,\ \ \ \ \forall k\in K \end{aligned}$$

29 $$\begin{aligned}&\begin{array}{l} w_{j}^{k}\ge w_{i}^{k}+{{q}_{i}}r_{ij}^{k}-Q(1-r_{ij}^{k}), \\ \forall j\in C\bigcup \{{{v}_{k}}\}\bigcup \{0\},i\in C\bigcup \{{{s}_{k}}\}\bigcup \{0\},k\in K \end{array} \end{aligned}$$

30 $$\begin{aligned}&w_{{{s}_{k}}}^{k}\le Q,\ \ \ \ \forall k\in K \end{aligned}$$

31 $$\begin{aligned}&\begin{array}{l} f_{i}^{k}+se{{r}_{i}}+{{t}_{ij}}r_{ij}^{k}-\alpha (1-r_{ij}^{k})\le f_{j}^{k}, \\ \forall i\in C\bigcup \{0\}\bigcup \{{{s}_{k}}\},j\in C\bigcup \{0\}\bigcup \{{{v}_{k}}\},k\in K \end{array} \end{aligned}$$

32 $$\begin{aligned}&U(f_{i}^{k})\ge SL,\quad i\in C_{S} k\in K \end{aligned}$$

33 $$\begin{aligned}&WT_{k}\le DT,\quad k\in K \end{aligned}$$

34 $$\begin{aligned}&f_{{{v}_{k}}}^{k}\ge f_{i}^{k}+se{{r}_{i}}+{{t}_{i{{v}_{k}}}}r_{i{{v}_{k}}}^{k}-\alpha (1-r_{i{{v}_{k}}}^{k}),\ \ \ \ i\in C,\nonumber \\&k\in K\ \ \ \ \end{aligned}$$

35 $$\begin{aligned}&f_{0}^{k}=0,\ \ \ \ k\in K \end{aligned}$$

36 $$\begin{aligned}&\sum \limits _{j\in \bigcup \{0\}}{{{x}_{ij}}}+\sum \limits _{h\in C\bigcup \{{{v}_{k}}\}}{\sum \limits _{k\in K}{r_{ih}^{k}}}\text {=1},\ \ \ \ \forall i\in C \end{aligned}$$

37 $$\begin{aligned}&{{x}_{ij}}\in \{0,1\},\ \ \ \ \forall (i,j)\in A \end{aligned}$$

38 $$\begin{aligned}&r_{ij}^{k}\in \{0,1\},\ \ \ \ \forall (i,j)\in A,k\in K \end{aligned}$$

39 $$\begin{aligned}&0\le {{y}_{i}}\le Q,\ \ \ \ \forall i\in C\bigcup \{0\} \end{aligned}$$

40 $$\begin{aligned}&0\le w_{i}^{k}\le Q,\ \ \ \ \forall i\in C\bigcup \{0\}\bigcup \{{{s}_{k}}\},k\in K. \end{aligned}$$

Formulas 16 and 17 are the flow constraints of professional vehicles. Formula 18 guarantees the fulfillment of demand at customer vertices. Formula 19 restricts the initial load level to the maximum capacity of a vehicle. Formulas 20 and 23 allow to determine the arrival time at node *j*. Formula 24 imposes a maximum number of available professional vehicles. Formulas 25 and 26 are the flow constraints of ODs’ routes. Formula 27 guarantees a limit on the number of available ODs. Formula 28 guarantees the number of departs from original node of occasional vehicle *k*. Formula 29 guarantees the fulfillment of demand at customer vertices in OD routes. Formula 30 restricts the initial load level to the maximum capacity of vehicle $k \in K$. Formulas 31–35 allow to determine the arrival time. Formula 36 guarantees that each customer is visited at most once, by either a professional vehicle or an occasional vehicle. Formulas 37–40 are ranges of decision variables.
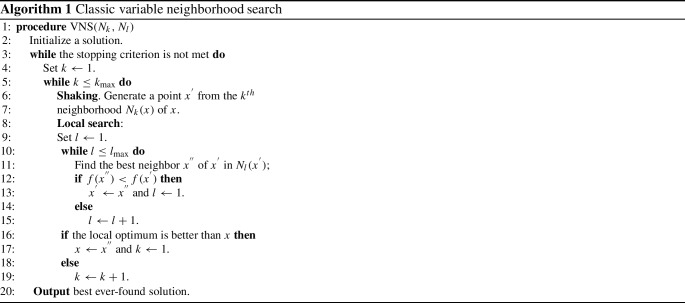

**Fig. 1:**
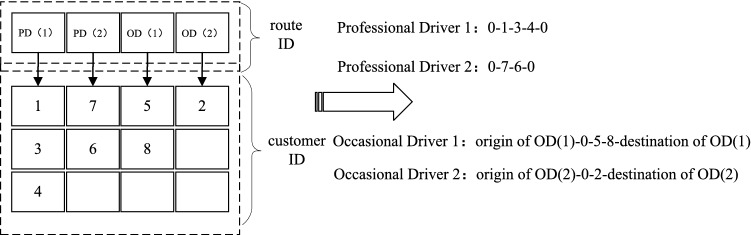
Encoding and decoding process

## Solution approach

### Variable neighborhood search (VNS)

Variable neighborhood search (VNS) was first proposed by Mladenovic and Hansen [31] to solve combinatorial and global optimization problems. VNS is derived from the idea of systematically changing neighborhoods during the search. In the classical implementation of VNS algorithm (Algorithm 1), four key components should be specified: (i) method to construct an initial solution; (ii) shaking method based on several neighborhood structures, which helps to slightly diversify the starting point of local search; (iii) local search, which improves the solution quickly, where meta-heuristic local search algorithm (i.e., SA, TS) and defined neighborhood operator can be used; and (iv) acceptance criterion that determines the termination of the search. $N_k$ and $N_l$ in Algorithm 1 are the neighborhood structure in shaking phase and local search, respectively.

However, there are some limitations in basic VNS: (i) the shaking solution generated randomly may lead to cycling phenomenon which means that the shaking solution may not be in a promising area where better solution can be found; (ii) information in elite solutions which is created in search process cannot be exploited; (iii) local search based on the size of neighborhood may not be a good way to in terms of efficiency.

In this paper, we propose an adapted version of VNS, inspired by the references above about VNS with learning and memory, to overcome the limitations of classic VNS. We name the adapted version of VNS in this paper as MVNS (memory-based variable neighborhood search).

### Memory-based variable neighborhood search

#### Solution representation and neighborhood structures

In MVNS, variable-size matrix is used to represent the solution of GVRP-PCPS (Fig. 1). The first line of matrix represents type (PD means professional driver, while OD means occasional driver) of routes as well as their numbers, and the other lines of matrix represents IDs of customers. Each column represents a feasible route, and customer in higher tier has higher priority to receive service. As is shown at the right side of decoding schematic diagram of Fig. 1, the decoded solution is composed of two professional routes and two occasional routes. For PDs, their origin and destination are both depot. For ODs, each of them has their own origin and destination.

In this paper, we use four operators to create neighborhood solutions, namely, Relocate, Swap, 2-OPT, and CrossExchange. Operators used in the following section are summarized in Table 5. Except for 2-Opt and CrossExchange, both Swap and Relocate have two versions which are used in single route and pair of routes, respectively.

**Fig. 2:**
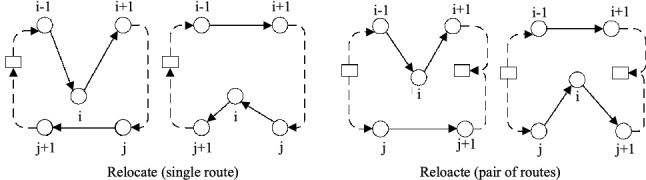
Relocate

The **Relocate** operator moves a customer from its position in one route to another position in either the same or a different route. We name the Relocate operator in single route as **Inner-relocate**, and the Relocate operator between two routes as **Outer-relocate**. Take Outer-relocate for instance in Fig. 2, the edges $(i-1,j)$, $(i,i+1)$, and $j,j+1$ are replaced by $(i-1,i+1)$, (*j*, *i*) and $(i,j+1)$, i.e., customer *i* from the origin route is placed after customer *j* in the destination route.

**Table 5:** Neighborhood operator used in LVNS

Operator	Pair of routes	Single route
Relocate	$\surd$	$\surd$
Swap	$\surd$	$\surd$
2-Opt		$\surd$
CrossExchange	$\surd$	

**Fig. 3:**
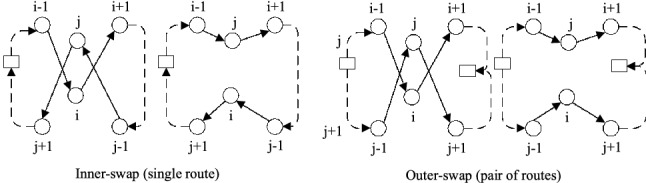
Swap

The **Swap** operator swaps two customers in single route or different routes. We name the Swap operator in single route as **Inner-swap**, and the Swap operator between two routes as **Outer-swap**. Take Outer-swap for instance in Fig. 3, the edges $(i-1,i)$, $(i,i+1)$, $(j-1,j)$, and $(j,j+1)$ are replaced by $(i-1,j)$, $(j,i+1)$, $(j-1,i)$, and $(i,j+1)$, i.e., two customers from different routes are simultaneously placed into the other routes.

**Fig. 4:**
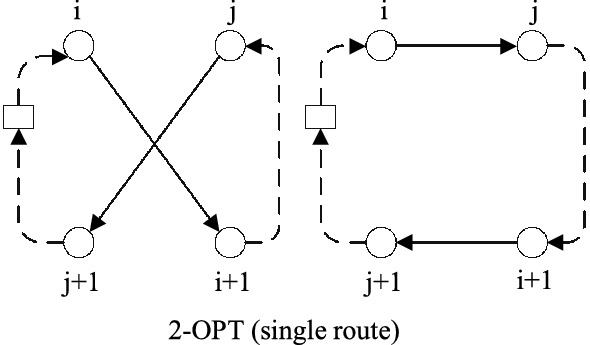
2-OPT

**2-Opt** tries to improve the tour using two edges to replace the other two edges. As is shown in Fig. 4, the edges $(i,i+1)$ and $(j,j+1)$ are replaced by edges (*i*, *j*) and $(i+1,j+1)$, thus reversing the direction of customers between $i+1$ and *j*.

**Fig. 5:**
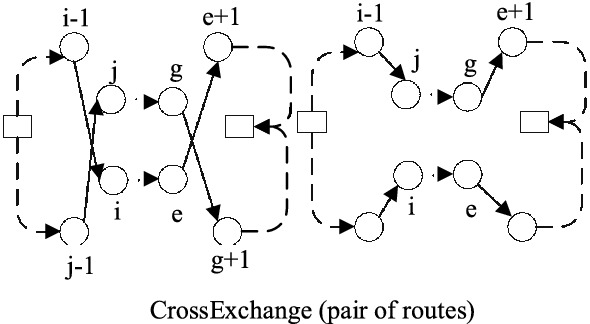
CrossExchange

**CrossExchange** tries to exchange two blocks in pair of routes to improve solution. Different from **Outer-swap**, the block in CrossExchange have a variable size. As is shown in Fig. 5, the block (*j*, ..., *g*) is created by breaking edge $(j-1,j)$ and $(g,g+1)$, while the block (*i*, ..., *e*) is created by breaking edge$(i-1,i)$ and $(e,e+1)$. Two updated tours are created by moving block in origin tour into the broken position of the other tour.

In the whole paper, operators used in shaking phase are Outer-relocate, 2-Opt, and CrossExchange, while those in local search phase are Inner-relocate, Inner-swap, Outer-relocate, and Outer-swap.

#### Initialize solution

The put forward insertion heuristic (PFIH) for inserting customers into a route for the VRPTW was introduced by Solomon (1987). It is an efficient method for computing the cost of inserting a new customer into the current route. The feasibility of inserting a customer into a route is checked by inserting the customer between all the nodes in the current route and then select the edge that has the lowest additional insertion cost. The feasibility check examines all the constraints including time windows and load capacity. Only feasible insertion will be accepted. When the current route is full without accepting any new customer, PFIH will start a new route and repeat the procedure until all the customers are routed. PFIH serves the role of constructing route configuration for VRPTW, which is an efficient method to obtain feasible solutions. Detailed information of PFIH is available in Solomon’s paper (1987). In this paper, seed customer is chosen according to value calculated by Formula 41. The candidate customer with least value means seed customer. And then, unrouted customers are inserted at the best feasible position of current route until the constraints are not met for all the unrouted customers

41 $$\begin{aligned} S{{C}_{c}}=-\alpha {{d}_{0c}}+\beta {{e}_{c}}+\gamma angl{{e}_{c}}, \end{aligned}$$

where ${{d}_{0c}}$ means the distance between depot and candidate customer *c*; ${{e}_{c}}$ means earliest service time of customer *c*; $angl{{e}_{c}}$ means the polar angle between customer *c* and last customer in the last route; $\alpha$, $\beta$, and $\gamma$ mean weights, respectively.
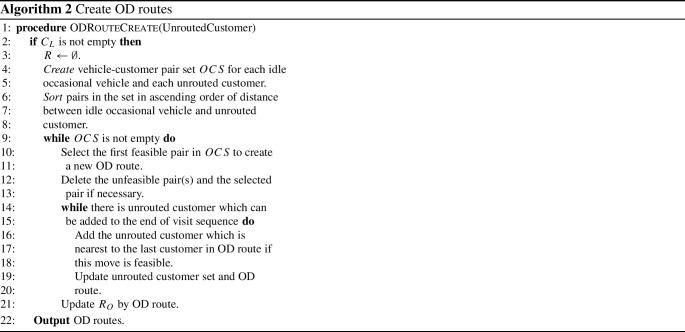


First, we use PFIH to create a set of candidate routes which are assigned to professional drivers. Because of the limited size of professional vehicles, additional routes are removed from set of candidate routes to make sure that its size is not larger than its limitation if necessary. Routes with lower load rate have lower priority to be kept in PD route set. If there are unrouted customers left, they can be assigned to feasible occasional routes. To give a concise description, we define several notations. Let ${{C}_{L}}$ the set of customers who have not been assigned to any vehicle. Let ${{R}_{O}}$ be the set of occasional routes. OD routes are created according to Algorithm 2.

#### Memory-based restart

The essence of the memory restart algorithm is the repetition and incomplete utilization of experience, which precisely provides ideas for the design of combinatorial optimization algorithms. Taillard et al. [32] first proposed a unified framework for adaptive memory programming. Subsequently, many scholars applied the idea of adaptive memory programming to solve vehicle routing problems. For more details on this methodology, readers could refer to the literature [33–35]. Learning mechanism is also used to improve many meta-heuristics, such as Ant Colony Optimization, Tabu Search. Thevenin [24] proposed an improved VNS with learning mechanism, where shaking solution was chosen according according to attractiveness of modified edges.

Inspired by the improvement approach based on memory and learning mechanism, we propose two kinds of methods to record specific attributes which were ever found in elite solution, namely, route-based method and edge-based method. We name route-based restart method as MVNS-r and name edge-based restart method as MVNS-e.

**MVNS-r**. OD route and PD route are recorded in the form of a four-tuple

$$<RouteType,Route,CoveredCustomerNum,Cost>$$.

*RouteType* means which type the recorded route is (OD route or PD route). *Route* means the specified route. *CoveredCustomerNum* means how many customers are covered by the related route. *Cost* means the cost if the related route is chosen. If the route is PD route, the cost includes fuel cost, carbon cost, and overtime wage. Total of compensation and carbon emission cost account for OD route cost.

To evaluate routes in memory, we define a lexicographic order for all the records. *RouteType* has priority over *CoveredCustomerNum* and *Cost*, while *CoveredCustomerNum* has priority over *Cost*. In practice, PD route is a better option for the sake of improving self-own vehicles’ usage rate, and CLP operators prefer PD route to OD route.

According to the partial order relationship of routes in memory, any route pair can be compared. Routes in new-found elite solution are used to update the memory with fixed size according to lexicographic order. It is worthy to note that, the better record is, the smaller its index is. At the very beginning of MVNS-r, initial solution is used to initialize memory structure.
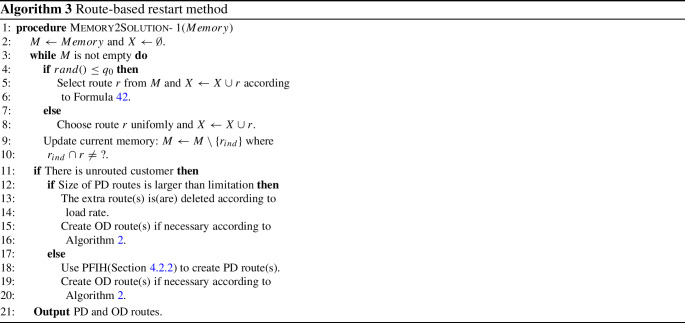


To enhance the diversity of solutions created by memory, we use both biased and uniform selection method [35]. If a random number *rand*() is not larger than greedy degree ${{q}_{0}}$, route is chosen from memory according to Formula 42; otherwise, route is chosen evenly

42 $$\begin{aligned} {{p}_{ind}}=\frac{2(I-ind+1)}{I(I+1)} \end{aligned}$$

where *I* is the number of records in memory. For biased method, sum of selection probability for each operator $\sum _{i n d=1}^{I} p_{i n d}$ is obviously equal to 1.

The pseudocode of route-based restart method is shown in Algorithm 3.

**MVNS-e**. Edge-based restart method is from the fact that elite solutions always have a good mix of common edges. Reasonable combination of these edges can improve the quality of restart solution, which helps VNS search in a promising area. To bias the random selection of edges in restart phase, we adapt the notion of move attractiveness to VNS [24]. The moves with higher attractiveness values have higher priority to be performed. The attributes modified by a move must be of course clearly identified.

##### Definition 1

Attributes of GVRP-PCPS. Attributes of GVRP-PCPS are characteristics related to the solution. In a complete solution of GVRP-PCPS, there are two types of routes, namely, PD route and OD route. For OD route, we merely consider the route with served customer(s). In this paper, attribute means one certain professional or occasional driver visits customer *i* and customer *j* in succession. We define the attribute as $<i,j{{>}_{P}}$ or $<i,j{{>}_{O}}$.

##### Definition 2

Weight of attribute. If attribute $<i,j>$ belongs to one improvement solution (now or before), there is a weight for this attribute $T{{r}^{t}}<i,j>$ at the iteration *t* which describes how good the attribute $<i,j>$ is based on historical experience. $T{{r}^{t}}<i,j>$ is updated by global optimal solution at *t* iteration $s^*(t)$ according to Formula 43. There is no weight for all the attributes at the very beginning of MVNS-e.

In the update process of attributes’ weight, those that are not in new global optimal solution but recorded in archive are decayed by coefficient $\rho$. At the same time, weights of attributes belong to both archive and ${{s}^{*}}(t)$ are enhanced. The increment of weights is as Formula 44. M is constant to control incremental degree

43 $$\begin{aligned}&T{{r}^{(t+1)}}<i,j>\leftarrow \rho T{{r}^{(t)}}(i,j)+\Delta T{{r}_{({s}^{*}(t))}}(i,j) \end{aligned}$$

44 $$\begin{aligned}&\triangle Tr^{\text {(t)}}<i,j>={\left\{ \begin{array}{ll} \frac{M}{f(s^{*}(t))} &{} <i,j>\,exists\\ &{} both\,in\,s^{*}(t)\,and\,archive\\ 0 &{} otherwise. \end{array}\right. }\nonumber \\ \end{aligned}$$

It can be seen that the strength of the attribute weight changes as the number of iterations increases: the weights of those attributes that often appear in the optimal solution of each iteration are relatively larger; those short-lived good attribute weight will gradually weaken, because they have not been strengthened.

In the whole optimization, once better solution is found, it can used to update edge-based memory according to Formula 43.

##### Definition 3

Solution attractiveness: Solution attractiveness means the total of weights of attributes in solution

45 $$\begin{aligned} A(s(t))=\underset{\text {<i,j>}\in s(t)}{\sum }Tr^{t}<i,j>. \end{aligned}$$

Thus, we can obtain the solution with the largest attractiveness in the candidate solution pool, using current solution and the selected operator, as is shown in Formula 46

46 $$\begin{aligned} s^{a}(t)=\underset{s'(t)\in pool(s(t),N)}{argmax}A(s'(t)), \end{aligned}$$

where *N* is the selected neighborhood operator, which is chosen randomly. The size of candidate pool is a constant.

The pseudocode of edge-based restart method is shown in Algorithm 4.
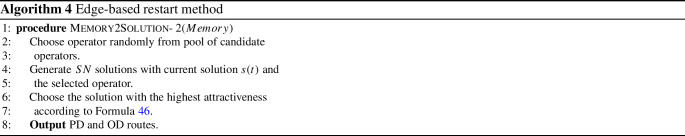


#### Framework of MVNS

In MVNS, once the ID of shaking neighborhood $k>k_{max}$, restart procedure will be executed according to Sect. “Memory-based restart”. $k_{max}$ means the number of neighborhoods used in shaking phase. 
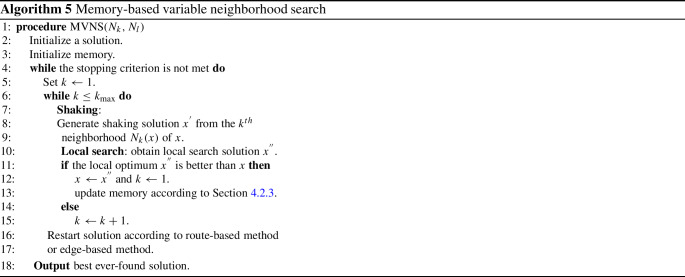

**Fig. 6:**
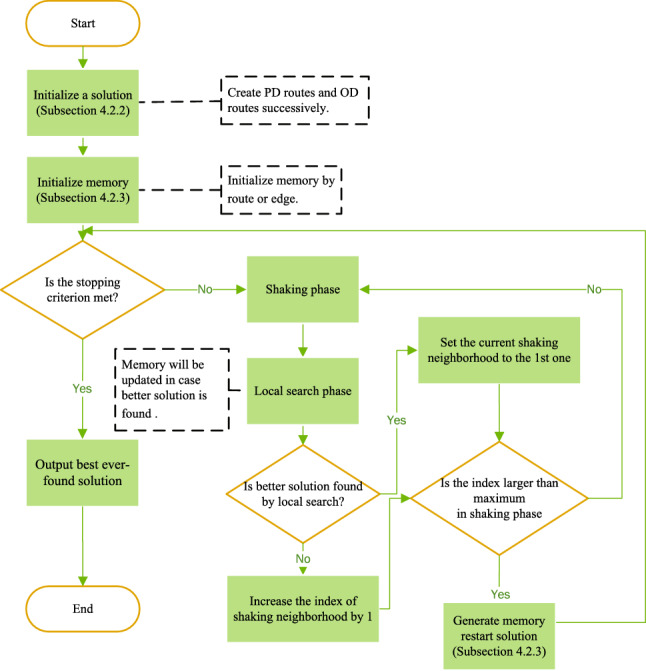
The flowchart of MVNS

*Shaking phase*. The order of neighborhood in shaking phase is Outer-relocate, 2-Opt, and CrossExchange. If better solution can not be found through local search phase whose initial solution is shaking solution created in $N_{k}$, $N_{k+1}$ will be selected if it exists.

*Local search phase*. The order of neighborhood in local search phase is Inner-relocate, Swap, and CrossExchange. Two acceptance strategies are common in local search, namely, first-accept (FA) and best-accept (BA). The first-accept strategy selects the first better neighbor that satisfies the constraints. The best-accept strategy examines all better neighbors satisfying the constraints and selects the best among them. In this paper, we use FA to improve the shaking solution to accelerate the optimization process.

*Restart phase*. Route-based restart method and edge-based restart are integrated into pseudocode of MVNS. As for MVNS-e, the neighborhoods used in edge-based restart phase are the same to those used in shaking phase.

The pseudocode of MVNS is shown in Algorithm 5. For a clear description of MVNS, a flowchart is also given for summarizing the process of the proposed algorithm and integrating the components.

## Instance generation

In this paper, we use two kinds of data set to assess the effectiveness of model and the proposed algorithm. The first data set is derived from Solomon’s VRPTW benchmark. To obtain the test instances for the GVRP-PCPS, given a VRPTW instance with customers whose coordinates $({{x}_{i}},{{y}_{i}})$, we randomly generated the origins and destinations for ODs, in the square with lower left hand corner $({{\min }_{i}}\{{{x}_{i}}\},{{\min }_{i}}\{{{y}_{i}}\})$ and upper right hand corner $({{\max }_{i}}\{{{x}_{i}}\},{{\max }_{i}}\{{{y}_{i}}\})$. The number of ODs is equal to the number of customers. To make more ODs available in crowdsourcing logistics system, we shrink the number of vehicles in original benchmark. The shrinking factors *SF* are set to 0.5.

**Table 6:** Parameters in MVNS

Component	Parameter	Description	Range	Value
Edge-based restart	*M*	*M* is a constant to control incremental degree of	5,10,15	10
		New-found better solution.(Formula 43)		
	$\rho$	$\rho$ is used to attenuate the attractive value of	0.85,0.9,0.95	0.85
		Attribute recorded.(Formula 44)		
	*SN*	*SN* is used to control the number of	40,50,60	50
		Shaking solutions.(Section “Memory-based restart”)		
Route-based restart	$q_0$	$q_0$ means greedy degree for using .	0.85,0.9,0.95 0.9	0.9
		(Section “Memory-based restart”)		
	*MZ*	*MZ* means the size of memory.	40,50,60	60
		(Section “Memory-based restart”)		
Other	*RM*	When there is no improvement within *RM*	5,10,15	15
		Outermost loops the algorithm will terminate. (Algorithm 5)		

The second data set is derived from a real-life city beer distribution situation. The latitude and longitude of the distribution center are (30.942738, 121.084699). 194 customers’ locations in Shanghai are known in the form of (*latitude*, *longitude*). Shortest distance between two locations is obtained from Gaode Map based on real road network, which is recorded in distance matrix. Origins and destinations of ODs are generated by the same means in the first test situation. To accelerate the process of simulation, distance between ODs’ location and customers’ locations or depot is calculated according to harversine formula, where earth radius is 6367 (km). As for real-life instance, the scheduled earliest delivery time $e_{i}$ of customer $i\in C$ is one random ranging from 0 to 600 (min), and the scheduled latest delivery time $l_{i}$ is one random ranging from $e_{i}$ to 600 (min). In both adapted benchmark instance and real-life instance, the latest service time of customer $i\in C$ is one random number sampled from range $\left[ l_{i},2*l_{i}\right]$ evenly, and sensitivity coefficient of customer $i\in C$ is created within $\left[ 0,2\right]$.

Other details of instances are shown in Appendix.

## Computational experiments

This section presents the results of our computational experiments. All computations were performed on an Intel Core i7-5700HQ CPU, with 2.60 GHz and 16 GB of RAM. The MVNS and the mathematical model were implemented in Matlab R2018a. We divided the computational study into two parts. The first part aims to assess the performance of the MVNS in terms of both efficiency and effectiveness. In the second part, the MVNS is tested on real-life instances.

### Benchmark test

There are six classes of instances in Solomon’s benchmark set, and we choose the first one of each class as the tested instance of that class, namely, C101, C201, R101, R201, RC101, and RC201. Because of the lack of parameters about GVRP-PCPS in Solomon’s benchmark, it is necessary to add some virtual parameters to origin benchmark instances, which is acceptable for the sake of tuning and component analysis. The related parameters of benchmark instances are shown in Appendix.

#### Algorithm parameter tuning

There are six main parameters in MVNS-r and MVNS-e. The descriptions about these parameters are shown in Table 6. An important step in adopting MVNS to solve real-life problems is to find appropriate parameters for it. Refer to [24, 35, 36], we give the range of each parameter, as is shown in Table 6. Taguchi experiments of $L_{9}(3^{3})$ (MVNS-r) and $L_{9}(3^{4})$ (MVNS-e) are conducted on six Solomon instances. Each experiment with certain parameter combination in Taguchi experiment table is repeated for 20 times. The value after tuning is shown in Table 6.

#### Component analysis

**Fig. 7:**
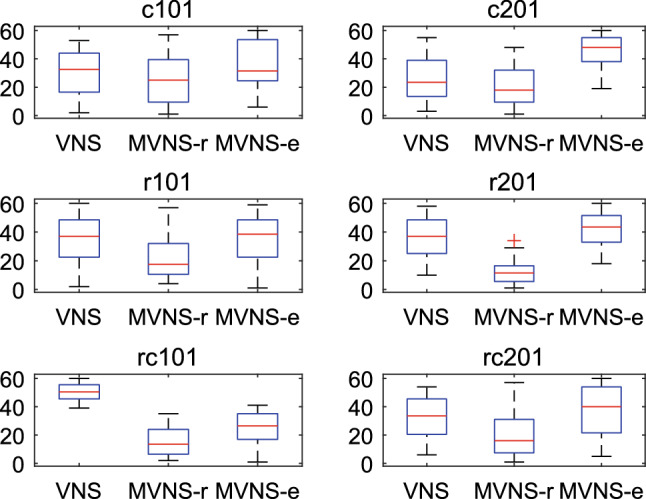
Box-plot of three versions of VNS

To validate effectiveness of the proposed memory restart mechanism in this paper, three versions of VNS are conducted, namely classic VNS (CVNS), MVNS-r, and MVNS-e. Each instance experiment for each version of VNS is repeated for 20 times. Sum of ranks among 60 solutions for each instance and each algorithm is recorded in Table 7. Statistical Friedman test is used to analyze the results above. Box-plot results are shown in Fig. 7. The p value of Friedman test is 0.0057, which indicates that there are obvious differences between the three algorithms. From the experimental results in Table 7 and Fig. 7, MVNS-r has advantage over the other two versions of VNS in terms of accuracy. Although MVNS-e shows poor performance in solving six benchmark instances, it does give a promising direction to design memory-based or learning-based algorithm, which has been proved by [24]. Shrinking the size of record in memory phase or usage of various operators in restart phase may be a good strategy to enhance edge-based restart method.

From the perspective of computational complexity, the proposed strategies in both MVNS-r and MVNS-e do not result in the increment of algorithm running cost. Based on the above analysis, we use MVNS-r to optimize real-life instances in Sect. 6.2.

**Table 7:** Rank comparation

Instance Set	CVNS	MVNS-r	MVNS-e
C101	587	**526**	717
C201	507	**407**	916
R101	683	**448**	699
R201	738	**255**	837
RC101	1008	**319**	503
RC201	648	**418**	764

### Real-life instance

In this section, the performance of the algorithm in terms of solution quality is tested with respect to different instance parameters, including minimal action cost ($R_{k}^{S}$), variable cost coefficient ($R_{k}^{V}$), compensation coefficient ($\lambda$), fuel price ($w_1$), and carbon tax price ($w_2$). For sensitivity analysis, the range of each parameter is given in Appendix. In Sect. 6.2, repeat times are 20 for each instance parameter setting, and mean value of each indicator is recorded.

#### Potential benefits of employing occasional drivers

**Fig. 8:**
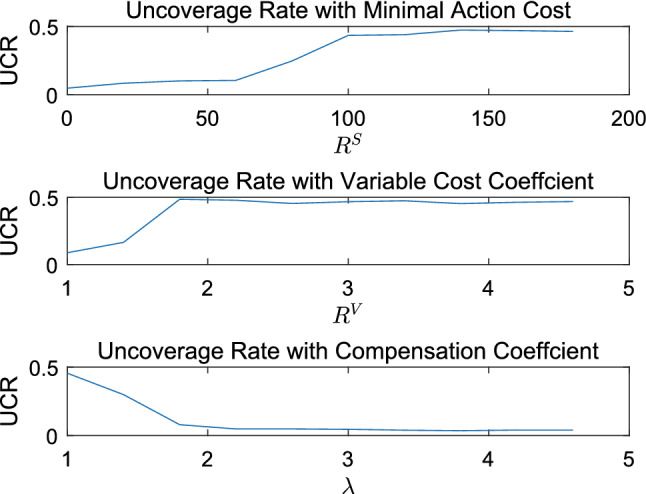
Potential benefits of employing ODs

Motivation of action for ODs is that the compensation obtained is higher than expectation. To validate the supply of ODs have positive effect on service, we take $R_{k}^{S}$, $R_{k}^{V}$, and $\lambda$ as independent variables, while the optimized objective *UCR* is controlled variable. From Fig. 8, we can conclude the more motivated the ODs are, the better *UCR* is. From the perspective of CLP owner, higher compensation is not cost-effective, because it cannot lead to significant improvement of *UCR* in a higher range of compensation coefficient.

**Fig. 9:**
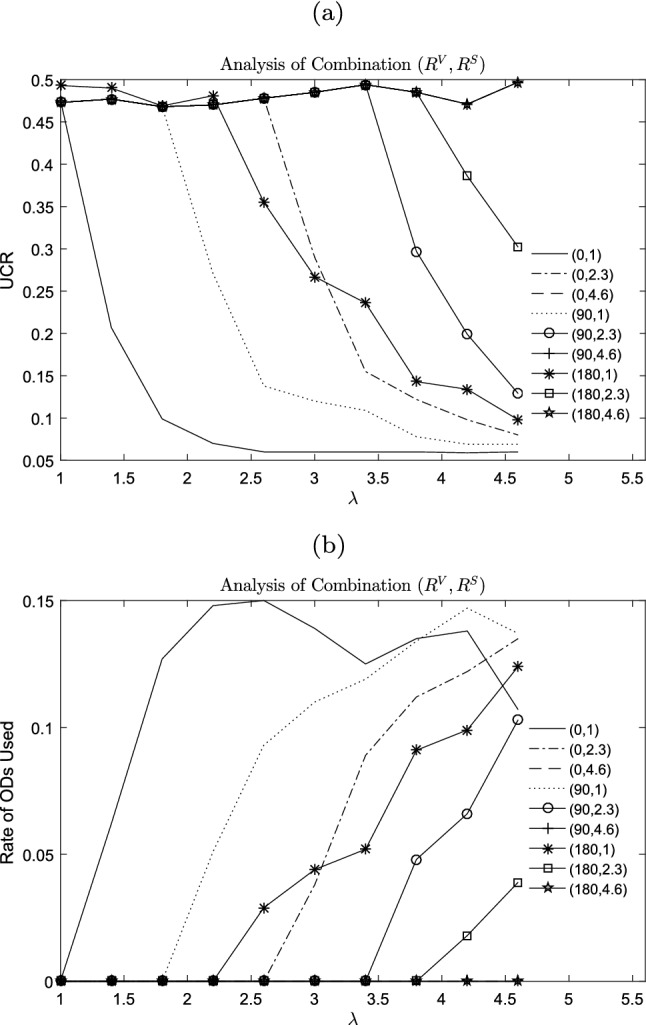
Analysis of combination

To analyze the effect of employing heterogeneous ODs on crowdsourcing logistics in this paper, we introduce nine kinds of passing-by drivers into this system, who are parameterized by ($R^S$,$R^V$). Results are shown in Fig. 9. As the the compensation coefficient increases, there is a downward trend of indicator *UCR*. However, passing-by drivers with higher minimal action cost and higher variable cost coefficient do not participate in crowdsourcing delivery task regardless of the given value of $\lambda$ ranging from 1 to 4.6. It is a significant management insight that differentiated pricing strategy for compensation can balance cost and *UCR* according to the characters of ODs.

#### The Influence of fuel price and carbon emission coefficient

**Fig. 10:**
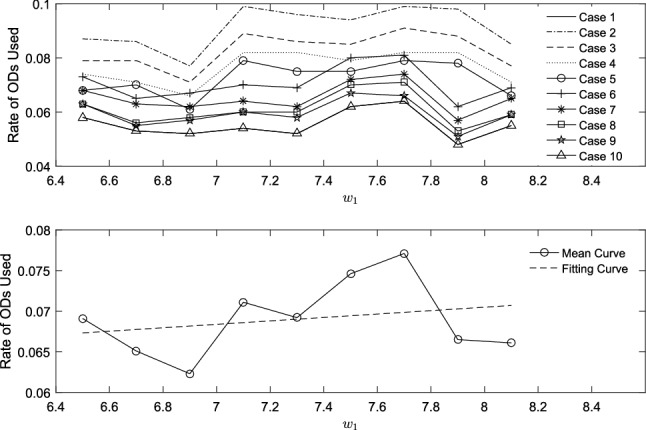
Rate of ODs used with fuel price

**Fig. 11:**
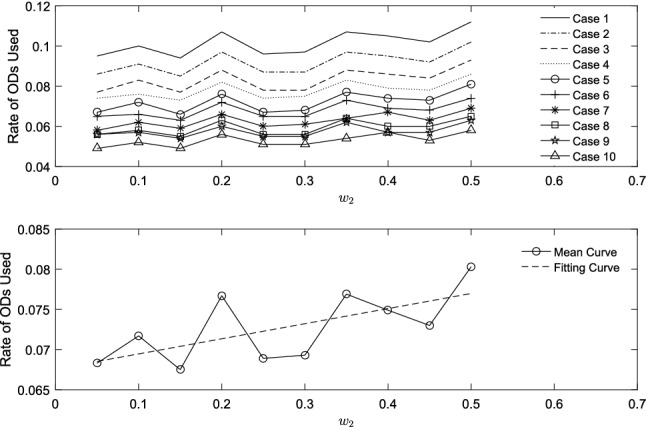
Rate of ODs used with carbon tax price

External market factors, such fuel price and carbon tax price, may give rise to change of operating strategy. In this section, we study how fluctuations in external market influence the usage of ODs under the same service level. To study intrinsical characteristic of problem in this section, we only consider two objectives, namely, *UCR* and operating cost ($TFC+TCEC+ODS+OW$). Case 1–Case 10 are same except for OD information. ODs are added to each case incrementally. Quantity of ODs in each Case $j\in [2,10]$ is $0.1\cdot (j-1)$ larger than Case 1. Figures 10 and  11 show how rate of ODs used changes as $w_1$ and $w_2$ increases. In both Figs. 10 and  11, picture above summarizes all the cases, while picture below includes mean curve of ten cases and its linear fitting line.

Compared to carbon market, sensitivity of ODs’ participation in fuel market is lower. It seems that choosing ODs become an option when carbon tax price increases, which reduces carbon emissions of PDs and adds a small amount of emissions related to detours. It will be quite important to make decision considering carbon trading, because saving means profitable.

**Fig. 12:**
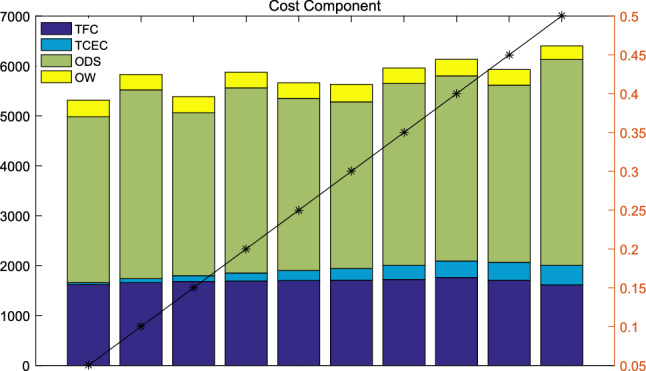
Cost Component with carbon tax price

To analyze cost changes with carbon tax price, operating cost component at different parameter level of $w_2$ is summarized in Fig. 12. The value of bar graph is shown at the left of Fig. 12, while stippled line shows the change of carbon tax price. Although the carbon tax price changes linearly, the change trend of operating cost is not obvious. In addition, TFC and ODS account for a large proportion at each level of $w_2$. Management insight from these results is that carbon tax has limited moderation for CLP owner if they focus too much on cost, and the government should take some other actions of carbon pricing.

**Fig. 13:**
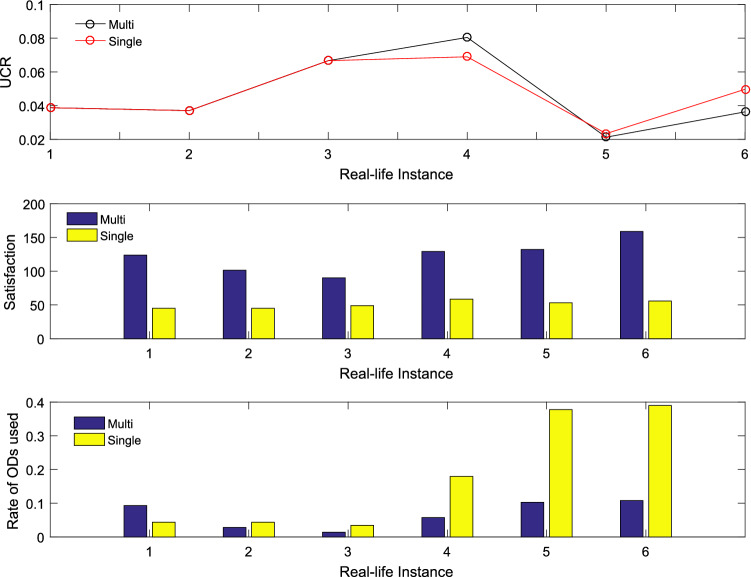
Analysis of delivery mode

#### Benefits of allowing multi-delivery

To validate the effectiveness of GVRP-PCPS (Multi) proposed in this paper, two compared models are used, namely, traditional delivery mode without ODs (Zero) and adapted version of GVRP-PCPS only allowing single delivery in OD route (Single). Results are shown in Table 8. Six real-life instances are generated randomly, which are named as RF01-RF06. Cost in Table 8 means operating cost, namely, the fourth optimized objective in Section “Problem description and formulation”. In Table 8, “Multi” delivery mode has advantage over the other two modes except for RF04 in terms of lexicographic objective function value. However, the difference between “Multi” and “Single” on RF04 is very small in terms of objective *UR*.

**Table 8:** Delivery mode comparation

Instance	Delivery mode	UCR	UR	UF	Cost
RF01	Zero	0.446	1.835	0.577	1406
	Single	0.072	1.877	0.780	2551
	**Multi**	0.072	1.901	0.835	2658
RF02	Zero	0.437	1.786	0.535	2240
	Single	0.037	1.890	0.596	4376
	**Multi**	0.03	1.920	0.573	3793
RF03	Zero	0.481	1.933	0.486	585
	Single	0	1.965	0.608	1029
	**Multi**	0	2.284	0.499	976
RF04	Zero	0.377	1.926	0.458	1894
	**Single**	0.066	1.951	0.729	3468
	Multi	0.066	1.929	0.678	3241
RF05	Zero	0.471	1.897	0.459	1669
	Single	0.050	1.860	0.798	3842
	**Multi**	0.050	1.870	0.642	3280
RF06	Zero	0.426	1.921	0.365	864
	Single	0.056	1.901	0.749	1969
	**Multi**	0.056	1.981	0.496	1771

To further validate the effectiveness of the proposed model, we evaluate this model from the perspective of ODs’ satisfaction and participation. ODs’ satisfaction means average compensation of all the employed OD routes. Participation can be described by the rate of ODs used in the previous statement. In Fig. 13, “Multi” and “Single” show the similar performance in terms of *UCR* on six real-life instances. However, ODs in “Multi” have higher satisfaction than that in “Single” . Higher satisfaction means more stable in crowdsourcing logistics. Although “Single” has advantage over “Multi” in terms of rate of ODs used, it is less acceptable than “Multi” in practice.

## Conclusions and future research

In this paper, we have proposed a novel VRP model under the situation of sharing economy from a comprehensive perspective. Interests of stakeholders in the system have been optimized hierarchically by memory-based variable neighborhood search algorithm. The strategies to enhance VNS have been proved to effective through component analysis, which has enriched the pool of algorithms for partially crowdsourcing logistics delivery mode. The model in this paper may be further utilized to improve performance of management considering given goal constraints and priorities. Furthermore, memory-based VNS in this paper can be adjusted to solve other VRPs with various objectives. Finally, experimental results of real-life beer delivery indicate the importance of differentiated pricing, and authority might encourage companies to reduce carbon emissions by more effective means, such as carbon quota mechanism and subsidies for alternative energy vehicles.

As a novel mode with characteristic of light asset, crowdsourcing logistics still face a series of problems. As for this study, there are some optimized objectives which can be improved hierarchically (e.g., service type and balance type ), while some objectives should be optimized simultaneously (e.g., cost type and balance type) from the perspective of practice. Thus, it is a tricky problem to handle these objectives. In addition, the number of objectives in crowdsourcing logistics is usually larger than three, which adds the difficulty of optimization. It may be a promising research direction to use methodology of many-objective optimization in solving problems related to crowdsourcing logistics.

For the sake of modeling easily, many parameters in this paper are static and homogeneous, such as characters of customers, arrival of ODs, and the type of vehicles. How to model these uncertainties is the key to improve value of mathematic model in engineering. Stochastic programming and behaviour science may narrow this gap between theory and application.

The need to reduce carbon emissions is growing globally, operation management of crowdsourcing logistics under the situation of carbon trading or market is less paid attention to. In the future, we will study crowdsourcing logistics with carbon trading to give a more eco-friendly solution.

## Appendix A: Benchmark test

In Solomon’s benchmark, distance between two nodes are equal to travelling time, which means that velocity of vehicle is 1. The added parameters are given in Table 9. BT and DT are equal to upper of depot’s time window in each instance.

**Table 9:** Parameters in benchmark test

Symbol	Description	Value
$R^S$	Minimal action cost	1
$R^V$	Variable cost coefficient	0.1
$\lambda$	Compensation coefficient	0.5
$w_1$	Fuel price	7
$w_2$	Carbon tax price	0.25
$w_3$	Overtime wage per unit time	6
$E_f$	Carbon emission conversion factor	2.37
$\mu _0$	Fuel consumption rate of empty vehicle	0.08
$\mu ^{\text {*}}$	Fuel consumption rate of vehicle	0.12
	With full capacity	
*Sal*	Basic salary per day	200
*SL*	Designed service level of CLP	0.9

**Table 10:** Information of products

Type	Length	Width	Height	Weight
	(cm)	(cm)	(cm)	(kg)
Type 1	40	17	12	7.92
Type 2	40	20	17	9
Type 3	27.5	20	17	6
Type 4	38.5	36.5	35.5	7.92
Type 5	27.5	20	12	3.96

**Table 11:** Parameter setting (a)

Analyzed parameters	Range	Step	Unit
$R_{k}^{S}$	[0,180]	20	Yuan
$R_{k}^{V}$	[1,4.6]	0.4	Yuan/km
$\lambda$	[1,4.6]	0.4	Yuan/km
$w_1$	[6.5,8.3]	0.2	Yuan/L
$w_2$	[0.05, 0.5]	0.05	Yuan/kg

$^*$ The reference range of $w_1$ is from www.globalpetrolprices.com

$$^{**}$$ The reference range of $$w_2$$ is from www.statista.com

**Table 12:** Parameter setting (b)

Analyzed parameters	Other parameters					
	$R_{k}^{S}$	$R_{k}^{V}$	$\lambda$	$w_1$	$w_2$	*SL*
$R_{k}^{S}$	\	1	2	7	0.05	0.9
$R_{k}^{V}$	20	\	2	7	0.05	0.9
$\lambda$	20	1	\	7	0.05	0.9
$w_1$	20	1	2	\	0.05	0.9
$w_2$	20	1	2	7	\	0.9

## Appendix B: Real-life instance

For each real-life experiment in Section “Real life instance”, chosen customers are generated randomly from candidate set of 194 customers. Quantity of each type is a random ranging from 1 to 10. Information about products is shown in Table 10. The travelling speed of vehicles in real-life instance is set to 50km/h.

Unchanged parameters are given:$w_3$(30 Yuan/h), $E_f$(2.37 kg/L)^1^, $\mu _{0}$(0.08 L/km), $\mu ^{\text {*}}$(0.12 L/km), *Sal*(200 Yuan), BT(8 h), DT(10 h). Analyzed parameters are given in Tables 11 and 12. Vehicle parameters in this paper are given referring to Jinbei. Maximum loading weight of vehicles is set to 2000 kg. In real life, maximum loading volume is another important constraint. In real-life instances, we add this volume constraint to GVRP-PCPS to make solution suitable for practice. Maximum loading volume of vehicles is set to 3.04 $\text {m}^3$.
